# Drone aggregation behavior in the social wasp *Vespula germanica* (Hymenoptera: Vespidae): Effect of kinship and density

**DOI:** 10.1038/s41598-020-64232-9

**Published:** 2020-04-28

**Authors:** Maité Masciocchi, Bárbara Angeletti, Juan C. Corley, Andrés S. Martínez

**Affiliations:** 1 Grupo de Ecología de Poblaciones de Insectos, IFAB – Instituto de Investigaciones Forestales y Agropecuarias Bariloche- (INTA - CONICET), Bariloche, Argentina; 2 Departamento de Ecología, Centro Regional Universitario Bariloche, Universidad Nacional Del Comahue, Bariloche, Argentina

**Keywords:** Ecology, Behavioural ecology, Invasive species

## Abstract

Inbreeding can have negative consequences on population viability because of the reduced fitness of the progeny. In general, most species have developed mechanisms to minimize inbreeding such as dispersal and kin avoidance behavior. In the eusocial Hymenoptera, related individuals typically share a common nest and have relatively short mating periods, this could lead to inbreeding, and because of their single–locus complementary sex determination system, it may generate diploid males that could result in infertile triploid progeny representing a cost for the colony. *Vespula germanica*, is an eusocial wasp that has invaded many parts of the world, despite likely facing a reduced genetic pool during the arrival phases. We ask whether male wasp display specific aggregation behavior that favors genetic diversity, key to reduce inbreeding. Through a set of laboratory experiments, we investigated the effects of drone nestmateship and density on the aggregation behavior of *V. germanica* drones. We show that drones avoid aggregating with their nestmates at all densities while non-nestmates are avoided only at high densities. This suggests that lek genetic diversity and density could be regulated through drone behavior and in the long run minimize inbreeding favoring invasion success.

## Introduction

Inbreeding (i.e., mating with genetically close relatives) can have negative consequences on population viability because of the effects on the fitness of the progeny. Although some species can tolerate and even favor inbreeding, many other tend to avoid it because inbred mating can increase homozygosity and the expression of deleterious alleles which in the long term, increases the population´s susceptibility to fast changing environments^1,2^. In the Hymenoptera, inbreeding may lead to the additional costs associated with the single-locus complementary sex determination system. This is because heterozygous individuals at the sex-determining locus develop into diploid females and hemizygotes develop into haploid males, but when inbred mating occur, males could develop into inviable sterile diploid individuals^3^. It´s important to note that even though diploid males can develop by chance under outbreeding conditions or due to the presence of a more prevalent allele in the population, inbred mating increase the frequency of diploid males^4^. These diploid males may give rise to infertile triploid broods, at a fitness cost for the colony, at the expense of workers and queens^5–7^. In eusocial insects (individuals that live in a colony with only some individuals capable of reproducing), related individuals share a common nest and have relatively short mating periods, thus can potentially suffer from increased probabilities of encounters with relatives, since densities of reproductives may be high near the nests^2,3,8^.

Because of the negative impacts of inbreeding, many species have evolved ways to prevent or reduce it^9,10^. Such mechanisms, can occur before or after copulation, and include among others, spatial and temporal segregation of opposite sexes, spatial aggregations, recognition of kin, mating with more than one individual (polyandry) and the preferential use of stored sperm^11–13^. Among these, spatial aggregations of males waiting for reproductive females to arrive is commonly observed in insects^14–16^. This is especially the case in species where females mate more than once and have short mating periods, therefore male aggregations need to be diverse^17^. This high genetic diversity appears crucial to avoid inbreeding because the high numbers of individuals in aggregations could decrease the probabilities of mating with relatives^13,15,18,19^. Males from different nests aggregating together offer females a genetically mixed population reducing the chances for mating with relatives even without kin recognition abilities^13^. For example, in *Apis* spp. aggregation of drones may facilitate and ensure the rapid mating of queens with as many drones as possible during their mating flights^20^.

*Vespula germanica* (Hymenoptera: Vespidae) is an eusocial vespid native to Europe and north of Africa, that in the last decades has invaded successfully many countries^21^. In Argentina, the species was observed for the first time in 1980 and since then, populations have expanded considerably^22,23^. In Patagonia, colonies are annual and are started by a single overwintered queen during spring. Once the workers mature, they take over the task of colony maintenance and feed larvae, being the queen only responsible for egg laying. At the end of the summer the colony begins to produce hundreds of new reproductive queens (gynes) and males (drones), which mate outside the nest during autumn. After this, drones die and reproductive females hibernate. The negative impact that *V. germanica* has on different productive and urban activities is significant, and despite being a pest in many parts of the world, the available management strategies are limited and therefore success is often limited^24^. Currently, toxic baits targeted at workers and mechanical destruction of nests are the main strategies used to control invasive populations^21^. Interestingly, population-management strategies directed at reproductive individuals are not available yet, probably due to the elusive reproductive behavior of the species and the short duration of the reproductive period.

Previous studies describing the mating behavior of social wasps have focused mainly on females, and suggest that mating occurs outside nests, mediated by a sexual pheromone produced by females, with mating occurring with one to several drones at random^8,25–27^. Additionally, field observations report loose aggregations of up to several hundreds of males around trees and shrubs^28,29^. Knowledge on the mating behavior of *V. germanica* may help understand how social insects deal with the potential negative effects of inbreeding and may be important as a basis for the development of management strategies for this invasive wasp. Our aim in this study was to establish under laboratory conditions, the effects of relatedness and density in drone aggregation behavior. Our working hypothesis is based on the assumption that due to the negative effects of inbreeding in hymenopterans, *V. germanica* drones will promote genetically-diverse aggregations. In order to promote such aggregations, we expect to find an avoidance threshold toward kin which will be lower (i.e. a lower number of drones) than the avoidance threshold toward non-kin.

## Results

**Figure 1:**
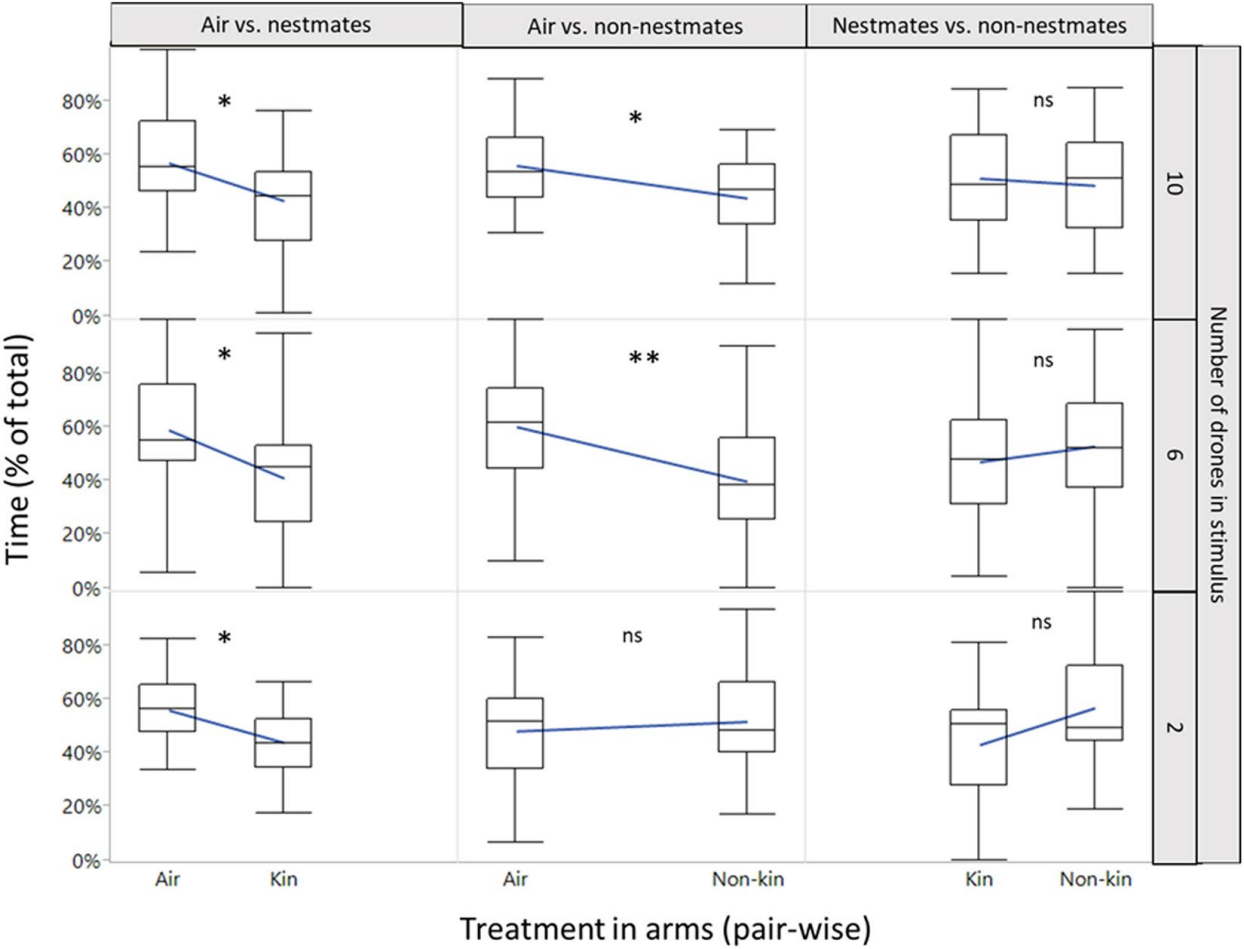
Time spent under different stimuli by *Vespula germanica* drones in a Peterson-type olfactometer under different treatments with variations of drone kinship level (i.e. nestmates and non-nestmates) and density (2, 6 and 10 individuals). The top and bottom boundaries of the boxes indicate 50% of the data spread. The line within the boxes marks the median value. Whiskers indicate the range of the data. Lines between boxes are the mean values. Asterisks denote significant differences between odour sources (paired Wilcoxon signed rank test: *=P < 0.05, **=P < 0.01) and “ns”, non-significant differences (P > 0.05). The line between treatments shows the trend between the data. R software version 3.6.1 (https://www.r-project.org) was used to create the figure.

We found no significant differences in the control trial in which we compared the time spent in the 4 arms containing humidified clean air only (χ^2^ = 3.8, P = 0.28, d.f.=3, n = 32). Response toward nestmates paired with clean air as the alternative, resulted in experimental drones spending more time in areas with clean air than those bearing nestmates volatiles, irrespective of their density (2 nestmates: W = −133, P = 0.005; 6 nestmates: W = −102, P = 0.02; 10 nestmates: W = −106, P = 0.017; Fig. 1). When 2 non-nestmates were presented *vs*. clean air, no significant differences were recorded (W = 7, P = 0.55), whereas when non-nestmates density was increased to 6 or 10 individuals, clean air was preferred over the non-nestmates (6 non-nestmates: W = −138, P = 0.004; 10 non-nestmates: W = −89, P = 0.048; Fig. 1). When nestmates and non-nestmates were presented simultaneously in the olfactometer, no statistical differences were detected under all densities (2 nestmates *vs*. 2 non-nestmates W = −73, P = 0.08; 6 nestmates *vs*. 6 non-nestmates W = 46, P = 0.2; 10 nestmates *vs*. 10 non-nestmates: W = −15, P = 0.38; Fig. 1). We found no statistical differences in the time spent in the central arena between treatments (χ^2^ = 5.7, P = 0.69, d.f.=8).

## Discussion

**Figure 2:**
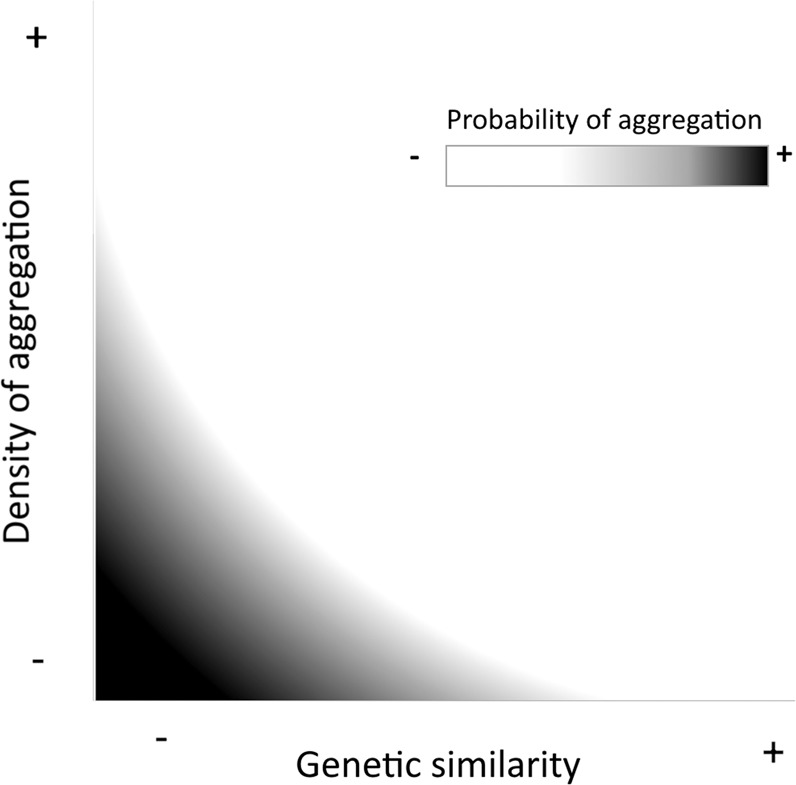
Hypothetical summary of the implications of the results of this study. Possible mechanism by which *Vespula germanica* drone behavior could promote genetic diversity in drone aggregations and maximize outbreeding. Figure 2 were created with paint.net (https://www.getpaint.net/) by ASM.

Our study was aimed at determining the existence of differential avoidance thresholds on the aggregation behavior of *V. germanica* kin and non-kin drones. Results indicate that drones avoid nestmates at all densities, while non-nestmates are avoided only at relatively high densities, suggesting that these two factors could be important in regulating *V. germanica* male aggregations, and could result in the conformation of leks with reduced densities of nestmates, therefore minimizing the probabilities of inbreeding. We hypothesize that drone aversive behavior toward kin may result in increased genetic heterogeneity of leks while regulating drone-density during aggregations (Fig. 2). Interestingly, no bias in behavior was observed when the choices offered consisted of nestmates vs. non-nestmates, but a trend to avoid nestmates was observed at the lowest density. This lack of statistical significance in nestmates *vs*. non-nestmates, could be due to the low contrast between the olfactory choices offered and the unnatural setting of the olfactometer. Past work has found that insect male aggregations in other hymenopterans are generally conformed by individuals of diverse genetic origin such as in the stingless bee *Scaptotrigona mexicana* (Hymenoptera: Apidae), were genetic studies indicate that aggregations are made up of males from 20 to 40 different colonies^30^. Also males of the eusocial stingless bee *Tetragonisca angustula* (Latreille) (Hymenoptera: Apidae) seem to congregate in mixed aggregations^13^, and males of *Scaptotrigona postica* aggregations were not related^17^. Genetic diversity in male aggregations facilitates encounters between unrelated mating partners, however a balance in the density of individuals within aggregations could be beneficial for both sexes, since too dense aggregations could result in difficulties to mate since the competition between males would obstruct and overwhelm females. For instance, males of tarantula hawk wasp, first monitor the density of competitors during mating, assess their relative size, age and kinship, and then decide whether to stay or move to another aggregation^15^.

Our results also indicate that kin recognition in *V. germanica* is based on chemical cues, since experimental subjects had no access to visual information from drones in the olfactometer chambers. The recognition of castes or relatives through cuticular lipids has been reported previously in insects^27,31^. Such recognition is based on chemical cues composed of a combination of colony-specific hydrocarbons present on both insect cuticles and nest surfaces^32,33^. Although these can be relatively large molecules with low volatilities, some of them can be perceived without tactile interaction at short distances, as established through behavioral studies on ants of the genus *Camponotus* and bumblebees, who can discriminate related from unrelated individuals from a short distance^34–36^. Having mentioned this, the relevance of visual cues in kin recognition cannot be ruled out, since their role in this aspect has been investigated and they can be important in sexual selection, mate attraction and mate choice^8,31,33,37^. For example, females of *Polistes simillimus* may evaluate male facial coloration during the choice of a sexual partner^38^. In addition, visual cues may be relevant in determining the distance between drones within an aggregation^20^.

It is worth mentioning that *V. germanica* females could further contribute to minimize inbreeding through mechanisms such as mate and sperm selection, but currently there is no detailed information on how these could contribute. Evidence of inbreeding avoidance regulated by females was found in the social wasp *Dolichovespula maculata* gynes^27^. Nevertheless, Goodisman *et al*. (2002) established that *V. germanica* queens can mate with more than one male, and this seems to happen randomly, with no evidence of females avoiding kin. With gynes not having a selective behavior toward potential mates, a mechanism to increase outbreeding, regulated by drones, would make sense. It has been recently suggested that dispersal in *V. germanica* is gyne-biased, while gynes have the potential of flying relatively large distances, drones fly relatively little, which implies remaining in the vicinity of the parental nest, where kin density is high^8,39^. This would suggest that, if no additional mechanism for increasing aggregation heterogeneity was in place, the risk of drone aggregations being conformed by a relatively high proportion of nestmates, would be high.

A better understanding of the factors that regulate the conformation of *V. germanica* leks could contribute to explain some of the drivers that lead to the invasion success observed in some yellow jacket species. When invasive species arrive to new territories, genetic homogeneity is expected until the arrival of new propagules^40^. This leads to hypothesis that on one hand female *V. germanica* reproductives would have high tolerance to mate with close relatives (as suggested by Goodisman *et al*. 2002) thus favoring the establishment of the species in the arrival phase when a high proportion of sibling drones are the only alternative to mate. Later, once new propagules arrive to the invasion area, increasing genetic increases, males would tend to aggregate with non-relatives and favor outbreeding. Such a mating system mechanism regulated by multiple factors, could favor outbreeding when heterogeneity is possible, but would also accept inbreeding, all regulated by gynes.

Detailed studies of male aggregations under natural conditions, considering factors such the factors that promote lek formation and their temporality, in addition to genetic studies to confirm the degree of relatedness within drone aggregations and surrounding nests, are warranted to fully understand the role of drone behavior of this successful invasive species. A better understanding of these mechanisms could be important to use as a basis for the development of management plans for the populations of this wasp, and other related invasive social wasps.

## Materials and Methods

### Insects

Males of *V. germanica* used in laboratory experiments were obtained from 10 dug-out nests removed from different locations at least 10 km apart in Bariloche, Argentina (previous studies report that genetic similarity declines with spatial distance, Goodisman *et al*., 2001^41^), during the onset of the reproductive period in the austral autumn (May 2018 and 2019). Subterranean nests were anesthetized with ethyl ether (98% purity; Sigma Aldrich, St. Louis, USA), excavated and immediately taken to the laboratory to separate drones in groups of ca. 50 individuals from the same nests in wire-mesh cages (10 ×10 ×10 cm). Cages were kept in controlled-temperature cabinets with 16 hours of light at 24 °C and 8 hours of darkness at 18 °C with *ad libitum* access to water, honey and pollen. Drones were used in bioassays after 9 ± 2 days of nest extraction, since previous studies in a similar species (*Vespula maculifrons* (Buysson) indicate that flight occurs when individuals are sexually mature^42^. In addition, preliminary trials in our laboratory (unpublished data) showed that *V. germanica* individuals are capable of flight as of 7 days after emergence.

### Bioassays

**Figure 3:**
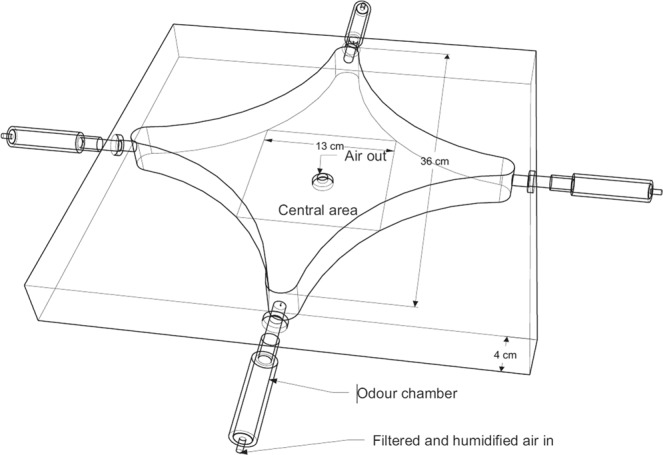
Four-arm olfactometer used in bioassays with *Vespula germanica* drones. Figure 3 were created with paint.net (https://www.getpaint.net/) by ASM.

The behavioral trails were performed in a controlled temperature room (24 °C) with artificial light. The olfactory response of drones was measured in a four-way olfactometer (Fig. 3) between 9 am and 4 pm. The arena, based on Pettersson^43^ and Vet *et al*.^44^, was made of polyamide (Grilon; Ems-Chemie, Domat/Ems, Switzerland) and consisted of four arms disposed symmetrically around a central area. The air flowed through the odor chambers, into each of the four arms and lastly through the central circular orifice at 0.5 l *per* min *per* arm. Air was previously filtered through 0.5 kg of activated charcoal and then humidified through 0.7 l of distilled water to prevent biases due to differences in humidity of odor sources^45^. Air flowed through polytetrafluoroethylene hoses (0.5 cm inner diameter) and we used brass fittings to connect them to the equipment. The arena was illuminated with white-light LEDs (2.5 m of LEDs strip) set in a circumference at 1.5 m over the olfactometer with 60 surface-mounted diodes per m (60 W; Alic, Buenos Aires, Argentina). Olfactory stimuli (live insects) were introduced into glass cylindrical chambers (2 cm diameter, 5 cm long) and were not visible from the inside the olfactometer.

Different number of live drones were placed in the glass chambers connected to the air stream that entered each of the olfactometer arms. The setup was used as a two-way olfactometer, with two odor sources presented simultaneously to individual wasps. The response toward different stimuli was tested for 32 drones (*i.e*., a total 32 replicates) for each treatment. For this, a single male was introduced in the central arena of the olfactometer and left for 1 min to acclimate, after which the experiment began allowing the wasp to move freely within the olfactometer during 5 min. After each replicate, the olfactometer was washed thoroughly with water and alcohol and left to dry. After four replicates, odor sources were renewed and in order to minimize orientation biases and possible left over non-polar compounds, their positions relative to the olfactometer arms interchanged according to a pre-defined rotation scheme.

Nine different treatments with variations in nestmateship and density were carried out: (a) *clean air vs. nestmates*: two empty glass cylindrical chambers (i.e., clean air) and two chambers containing 2, 6 or 10 drones from the same nest; (b) *clean air vs. non-nestmates*: two empty chambers and two chambers containing 2, 6 or 10 drones from nests separated by at least 10 km from the site that the experimental subjects´ nest was removed; and (c) *nestmates vs. non-nestmates*: two chambers containing in total 2, 6 and 10 drones from the same nest as the experimental subject, and two chambers containing the same number of drones from unrelated nests. For each of the 32 replicates carried out for each treatment, experimental subjects were obtained from at least four different nests and each focal male was used only once.

### Data analysis

Drone movement in the olfactometer was recorded as an *.avi* file at five frames/second using a web camera (Genius FaceCam 1000; KYE Systems, New Taipei City, Taiwan) during the 5 min the experiment lasted. The position (X,Y coordinates) of the wasp was determined in each frame via analysis with ImageJ^46^ in combination with the wrMTrck plugin^47^. Once the wasps´ position was determined at each frame, the percentage of time spent in each stimulus area (i.e., clean air, kin or non-kin) was determined, in addition to the time spent at the central area (a 13 cm sided square, centered in the olfactometer).

Because normality assumptions were not met, the responses of drones toward the different stimuli were contrasted with paired Wilcoxon rank sum tests. Contrasts were carried out under the hypothesis that time spent under the kin-area of the olfactometer would be significantly less than that spent in its alternative area. When contrasting responses containing non-nestmates as stimuli, we expected them to be biased toward these. For each replicate, paired responses were considered to be the time spent in each of the two different stimuli areas. We carried out an additional Wilcoxon test to compare the time (in percentage) spent in the central arena between treatments, expecting no differences between them, since we assumed that the time dedicated to exploration (i.e. time spent in the central arena evaluating) would not be different between the nine different treatments. Before the behavioral assays were carried out, we run an additional assay to ensure that no biases occurred in the olfactometer due to differences in the arms, factors such as position, lighting conditions, construction, air-flow speed, etc. For this, we did a control trial with the 4 arms running humidified clean air only. All analyses were performed using R software v.3.6.1 (R Development Core Team, 2019).

## References

[1] Keller L, Waller D (2002). Inbreeding effects in wild populations. Trends Ecol. Evol..

[2] Tabadkani SM, Nozari J, Lihoreau M (2012). Inbreeding and the evolution of sociality in arthropods. Naturwissenschaften.

[3] Van Wilgenburg E, Driessen G, Beukeboom LW (2006). Single locus complementary sex determination in Hymenoptera: An ‘unintelligent’ design?. Front. Zool..

[4] Ross KG, Fletcher DJC (1986). Diploid male production - a significant colony mortality factor in the fire ant Solenopsis invicta (Hymenoptera: Formicidae). Behav. Ecol. Sociobiol..

[5] Heimpel GE, de Boer JG (2008). Sex Determination in the Hymenoptera. Annu. Rev. Entomol..

[6] Whitehorn PR, Tinsley MC, Brown MJ, Darvill B, Goulson D (2009). Impacts of inbreeding on bumblebee colony fitness under field conditions. BMC Evol. Biol..

[7] Bogo G (2018). No evidence for an inbreeding avoidance system in the bumble bee Bombus terrestris. Apidologie.

[8] Martínez AS, Masciocchi M, Pisman N, Villacide JM, Corley JC (2018). Mate-searching behavior in the invasive German wasp, Vespula germanica, in Patagonia. Entomol. Exp. Appl..

[9] Pusey A, Wolf M (1996). Inbreeding avoidance in animals. Trends Ecol. Evol..

[10] Tsutsui N, Suarez A, Holway D (2000). Reduced genetic variation and the success of an invasive species. Proc. Natl. Acad. Sci. USA.

[11] Bretman A, Wedell N (2004). Molecular evidence of post–copulatory inbreeding avoidance in the field cricket Gryllus bimaculatus. Proc. R. Soc. London. Ser. B Biol. Sci..

[12] Blomquist, G. & Bagnères, A. *Insect hydrocarbons: biology, biochemistry, and chemical ecology*. (Cambridge University Press, 2010).

[13] Dos Santos CF, Francisco F, de O, Imperatriz-Fonseca VL, Arias MC (2016). Eusocial bee male aggregations: Spatially and temporally separated but genetically homogenous. Entomol. Exp. Appl..

[14] Dos Santos CF, Ferreira-Caliman MJ, Nascimento FS, Bloch G (2015). An Alien in the group: Eusocial male bees sharing nonspecific reproductive aggregations. J. Insect Sci..

[15] Thornhill, R. & Alcock, J. *The evolution of insect mating systems*. (Harvard University Press, 1983).

[16] Sivinski, J. & Petersson, E. Mate choice and species isolation in swarming insects. In *The evolution of mating systems in insects and arachnids* (eds. Choe, J. & Crespi, B.) (Cambridge University Press, 1997).

[17] Paxton R (2005). Male mating behaviour and mating systems of bees: an overview. Apidologie.

[18] Ayasse M, Paxton RJ, Tengo J (2001). Mating behavior and chemical communication in the order Hymenoptera. Annu. Rev. Entomol..

[19] Shaw AK, Kokko H (2014). Mate finding, Allee effects and selection for sex-biased dispersal. J. Anim. Ecol..

[20] Koeniger N, Koeniger G, Gries M, Tingek S (2005). Drone competition at drone congregation areas in four Apis species. Apidologie.

[21] Beggs JR (2011). Ecological effects and management of invasive alien Vespidae. BioControl.

[22] Willink A (1980). Sobre la presencia de Vespula germanica (Fabricius) en la Argentina (Himenóptera: Vespidae). Neotrop. La Plata.

[23] Masciocchi M, Corley JC (2013). Distribution, dispersal and spread of the invasive social wasp (Vespula germanica) in Argentina. Austral Ecol..

[24] Dimarco RD, Masciocchi M, Corley JC (2017). Managing nuisance social insects in urban environments: an overview. Int. J. Pest Manag..

[25] Goodisman MAD, Matthews RW, Crozier RH (2002). Mating and reproduction in the wasp Vespula germanica. Behav. Ecol. Sociobiol..

[26] Brown RL, El-Sayed AM, Suckling DM, Stringer LD, Beggs JR (2013). Vespula vulgaris (Hymenoptera: Vespidae) gynes use a sex pheromone to attract males. Can. Entomol..

[27] Derstine NT, Ohler B, Jimenez SI, Landolt P, Gries G (2017). Evidence for sex pheromones and inbreeding avoidance in select North American yellowjacket species. Entomol. Exp. Appl..

[28] Spradbery, J. P. *Wasps: an account of the biology and natural history of solitary and social wasps. Biology* (University of Washington Press, 1973).

[29] Greene, A. Dolichovespula and Vespula. In *The Social Biology of Wasps* 263–305 (1991).

[30] Mueller MY, Moritz RFA, Kraus FB (2012). Outbreeding and lack of temporal genetic structure in a drone congregation of the neotropical stingless bee *Scaptotrigona mexicana*. Ecol. Evol..

[31] Cappa F, Beani L, Cervo R (2016). The importance of being yellow: Visual over chemical cues in gender recognition in a social wasp. Behav. Ecol..

[32] Gamboa GJ (2004). Kin recognition in eusocial wasps. Ann. Zool. Fennici.

[33] de Souza AR (2017). No Evidence of Intersexual Kin Recognition by Males of the Neotropical Paper Wasp Polistes versicolor. J. Insect Behav..

[34] Brandstaetter AS, Endler A (2008). Nestmate recognition in ants is possible without tactile interaction. Naturwissenschaften.

[35] Whitehorn P, Tinsley M, Goulson D (2009). Kin recognition and inbreeding reluctance in bumblebees. Apidologie.

[36] Beani, L. *et al*. Cuticular hydrocarbons as cues of sex and health condition in Polistes dominula wasps. *Insectes Soc*. 10.1007/s00040-019-00721-z (2019).

[37] Cervo, R., Cini, A. & Turillazzi, S. Visual recognition in wasps. In *Social recognition in invertebrates* (eds. Aquiloni, L. & Tricarico, E.) (Springer International Publishing, 2015).

[38] de Souza A, Mourão CA, Do Nascimento FS, Lino-Neto J (2014). Sexy faces in a male paper wasp. PLoS One.

[39] Masciocchi M, Martínez AS, Pereira AJ, Villacide JM, Corley JC (2016). Dispersal behavior of yellowjacket (Vespula germanica) queens. Insect Sci..

[40] Eyer PA (2018). Inbreeding tolerance as a pre-adapted trait for invasion success in the invasive ant Brachyponera chinensis. Mol. Ecol..

[41] Goodisman, M. A. D., Matthews, R. W., Spradbery, J. P., Carew, M. E. & Crozier, R. H. Reproduction and Recruitment in Perennial Wasp Vespula germanica. *J. Hered*. 346–349 (2001). 10.1093/jhered/92.4.346 11535648

[42] Ross KG (1983). Laboratory studies of the mating biology of the eastern yellowjacket, Vespula maculifrons (Hymenoptera: Vespidae). J. Kansas Entomol. Soc..

[43] Pettersson J (1970). An aphid sex attractant. Insect Syst. Evol..

[44] Vet LEM, Lenteren JC, van, Heymans M, Meelis E (1983). An airflow olfactometer for measuring olfactory responses of hymenopterous parasitoids and other small insects. Physiol. Entomol..

[45] Martínez AS, Hardie J (2009). Hygroreception in olfactometer studies. Physiol. Entomol..

[46] Schneider CA, Rasband WS, Eliceiri KW (2012). NIH Image to ImageJ: 25 years of image analysis. Nat. Methods.

[47] Nussbaum-Krammer C, Neto M, Brielmann R, Pedersen J, Morimoto R (2015). Investigating the spreading and toxicity of prion-like proteins using the metazoan model organism C. elegans. J. Vis. Exp..

